# Development of the B cell cancer vaccine HER-vaxx for the treatment of her-2 expressing cancers

**DOI:** 10.3389/fonc.2022.939356

**Published:** 2022-12-12

**Authors:** Nicholas J. Ede, Anthony J. Good, Joshua Tobias, Erika Garner-Spitzer, Christoph C. Zielinski, Ursula Wiedermann

**Affiliations:** ^1^ Immunotherapy R&D Department, Imugene Limited, Sydney, NSW, Australia; ^2^ Institute of Specific Prophylaxis and Tropical Medicine, Center for Pathophysiology, Infectiology and Immunology, Medical University of Vienna, Vienna, Austria; ^3^ Central European Cancer Center, Wiener Privatklinik, and Central European Cooperative Oncology Group (CECOG), Vienna, Austria

**Keywords:** cancer vaccine, peptide, immunotherapy, HER-2, PD-1

## Abstract

Her-2/neu is a tumor-associated protein that is overexpressed in a number of malignancies, including advanced cancer of the stomach, and has been proposed as a human cancer vaccine target. Overexpression of Her-2/neu in human breast and gastric carcinomas correlates with a more aggressive course of disease that results in poorer overall survival rates and shorter times to disease progression than in patients with tumors without overexpression of Her-2/neu. Cancer vaccines have the ability to stimulate the native immune system and in particular engineered B cell epitopes can elicit high affinity polyclonal antibodies with similar efficacy to Her-2 monoclonal antibodies such as trastuzumab (Roche). HER-Vaxx is under development as a therapeutic B cell vaccine for the treatment of gastric cancer in patients with Her-2/neu overexpressing metastatic or advanced adenocarcinoma of the stomach or gastroesophageal junction, referred to as advanced cancer of the stomach. P467-CRM197, the vaccine’s immunogenic component, contains a single peptide antigen composed of 3 individual linear B cell epitope peptide sequences selected from the oncoprotein Her-2/neu that induce the patient’s own B cells to produce endogenous anti-Her-2/neu antibodies. This review provides results from comprehensive preclinical studies encompassing primary and secondary pharmacodynamics, biodistribution and safety studies. These studies were performed to support clinical development of HER-Vaxx. Results from the GLP toxicology study in rodents showed that the vaccine did not produce any observable adverse effects suggesting that the doses proposed for the clinical trial should be well tolerated in patients.

## Background

### Her-2/neu, tumors and current treatment

The transmembrane tyrosine kinase receptor Human Epidermal Growth Factor Receptor 2 (Her-2/neu) is a member of the epidermal growth factor receptor (EGFR/ErbB) family. When overexpressed, Her-2/neu is typically the primary driver of proliferation for the malignant cells. Her-2/neu has been found to be overexpressed in about 10% to 20% of several cancer types, in breast cancer in particular but also in 10% to 20% of patients with gastric cancer. Her-2/neu overexpression is associated with a worse prognosis, more aggressive disease, and poorer survival. The monoclonal antibody trastuzumab (Herceptin^®^, Roche) targets Her-2/neu) was successfully introduced in 1998 for first-line (1L) metastatic disease for Her-2-positive breast cancer and in 2010 for Her-2-positive advanced or metastatic gastric or gastroesophageal junction cancer (GC/GEJ cancer) (1–5).

Trastuzumab affects tumor growth by both direct and indirect mechanisms, recently reviewed (6). The direct mechanism involves the binding of antibodies to Her-2, which alters the receptor signaling properties leading to growth inhibition (inhibition of proliferation). The indirect mechanism involves complement-dependent cytotoxicity (CDC) and antibody-dependent cell-mediated cytotoxicity (ADCC).

Gastric cancer (GC) is one of the most common cancers worldwide and the third leading cause for cancer death. About 10 to 20% of GC are Her-2 over-expressing (1). Gastric cancer is often diagnosed at an advanced stage, defined as unresectable locoregional or metastatic disease, which has extremely poor prognosis with 5-year survival not exceeding 5–20% (Seers Cancer factsheets). Despite significant progress in the treatment of metastatic GC, most patients with metastatic disease succumb to the disease. Therefore, improving the treatment options and effectiveness is critical in changing the outcomes for patients with advanced and metastatic gastric cancer.

Recent advances in treatment of Her-2/neu-overexpressing GC/GEJ cancer led to the approvals for Enhertu^®^ (January 2021) in patients who have progressed on trastuzumab containing treatment and Keytruda^®^ (May 2021) as 1L treatment in combination with trastuzumab and chemotherapy in patients with HER2/neu-overexpressing advanced or metastatic GC/GEJ cancer (7, 8). Trastuzumab-deruxtecan (Enhertu^®^) is the first targeted agent to receive approval in advanced or metastatic HER2-overexpressing GC/GEJ cancer patients who retain their Her-2/neu overexpression after progression on 1L trastuzumab-containing treatment. Enhertu^®^ is an antibody-drug conjugate consisting of the humanized monoclonal antibody trastuzumab covalently linked to the topoisomerase I inhibitor deruxtecan (8). A maintenance treatment with trastuzumab, or other anti-Her-2/neu-targeted agent, alone after patients terminate the Enhertu^®^ “conjugate,” is not foreseen in this approval.

First-line treatment with pembrolizumab confirms the upregulation of PD-L1 in Her-2-expressing gastric cancer that has previously been described as “sensitive to treatment with anti-PD-L1 antibody” (9). The recent approval in advanced or metastatic HER2/neu-overexpressing GC/GEJ cancer is not limited by patients’ tumor combined positive score (CPS), or microsatellite instability (MSI) or PD-L1 expression. PD-L1 expression is equally observed in Her-2 positive and negative patients as investigated in a matched case control study, and the dual inhibition of an anti-Her-2 agent and a checkpoint-inhibitor has been discussed (10). The Keynote811 study has investigated this strategy and was basis for the accelerated approval of Keytruda^®^ in 1L advanced or metastatic GC/GEJ cancer (7).

In contrast, approval of Keytruda^®^ in 3L GC/GEJ cancer patients with an CPS score >1 was revoked in 2021 in the US due to lack of overall survival benefit despite the previous accelerated approval based on objective response rate (ORR) in 2017 (11). It appears that for Her-2/neu-overexpressing GC/GEJ cancer targeting upregulation of PD-L1 at the same time as Her-2/neu is a potentially effective treatment strategy.

There remains an unmet need for Her-2/neu-overexpressing GC/GEJ patients for treatments that are well tolerated, induce durable response, overcome treatment resistance, potentially offer alternative treatment to chemotherapy, and ultimately extend overall survival.

### Strategies to overcome resistance to immunotherapy: B cell cancer vaccines

Resistance to immunotherapy treatments such as trastuzumab in gastric cancer patients has been seen by the loss of Her-2 expression post treatment (12) and the upregulation of PDL1 leading to escape mechanism and progression (13, 14).

To overcome resistance to immunotherapy within Her-2/neu-overexpressing GC/GEJ cancer, one promising strategy is to increase the number of cytotoxic immune cells within the tumor microenvironment *via* the use of polyclonal vaccines such as HER-Vaxx (IMU-131). Active immunization with HER-Vaxx (IMU-131) has induced high and long-lasting antibody levels and expanded lymphocyte subpopulations, such as interferon gamma (IFNγ)-producing CD4 and CD8 T cells (15, 16).

B cell cancer vaccines and the active immunization strategy, where the patients become their own “antibody generating factory” have been extensively reviewed recently by both the Wiedermann (17) and Kaumaya (18) research groups. Polyclonal antibodies generated *via* active immunization are more durable with a constant level of antibody generated as long as the patient is dosed approximately every 3 months. In addition, with booster immunizations scheduled as part of the clinical protocol, the possibility of immunological memory exists. Safety is a primary advantage with no dose limiting toxicities (DLT’s) and HER-Vaxx related adverse events reported (12). The Kaumaya group has developed B-Vaxx and tested it in a Phase 1 trial (19). Whilst the mechanism of action of both vaccines is similar, HER-Vaxx generates antibodies from three separate B cell epitopes of the Her-2/neu receptor rather than two epitopes for B-Vaxx. This review focuses more on the development and evolution of the Wiedermann Group’s Her-2/neu vaccine HER-Vaxx, currently in clinical development by the immuno-oncology biotechnology company Imugene Limited (see www.imugene.com).

HER-Vaxx is expected to have a mechanism of action (MoA) similar to that of trastuzumab. As reported in the Phase 1b trial of HER-Vaxx (12), it was found that the generated polyclonal antibodies in several patients sera could inhibit intracellular Her-2 phosphorylation *in vitro*, suggesting that HER-Vaxx generated antibodies might have similar functionality as trastuzumab. In addition, HER-Vaxx immunization also induced cellular immune responses, as indicated for example, by production of predominantly Th1-biased CRM197- and Her-2-specific cytokines (15, 16). The aim of the therapeutic vaccine strategy is to complement or replace the high-dose passive immunization induced by trastuzumab with the patient’s own natural and long-lasting humoral response to Her-2/neu after immunization with the dosage form, HER-Vaxx.

To support the clinical testing of HER-Vaxx (IMU-131), this review details a description of B cell vaccine HER-Vaxx and comprehensive preclinical pharmacology, safety pharmacology and toxicology programs. The findings suggest that P467-CRM197 (IMU-131 administered IM as HER-Vaxx) has acceptable nonclinical pharmacology and safety profiles. This nonclinical safety profile is considered sufficient to support the clinical development of P467-CRM197 (IMU-131 administered IM as HER-Vaxx) as a therapeutic cancer vaccine for treatment of patients with advanced stomach cancer.

### Description of the B cell cancer vaccine HER-Vaxx

**Figure 1:**
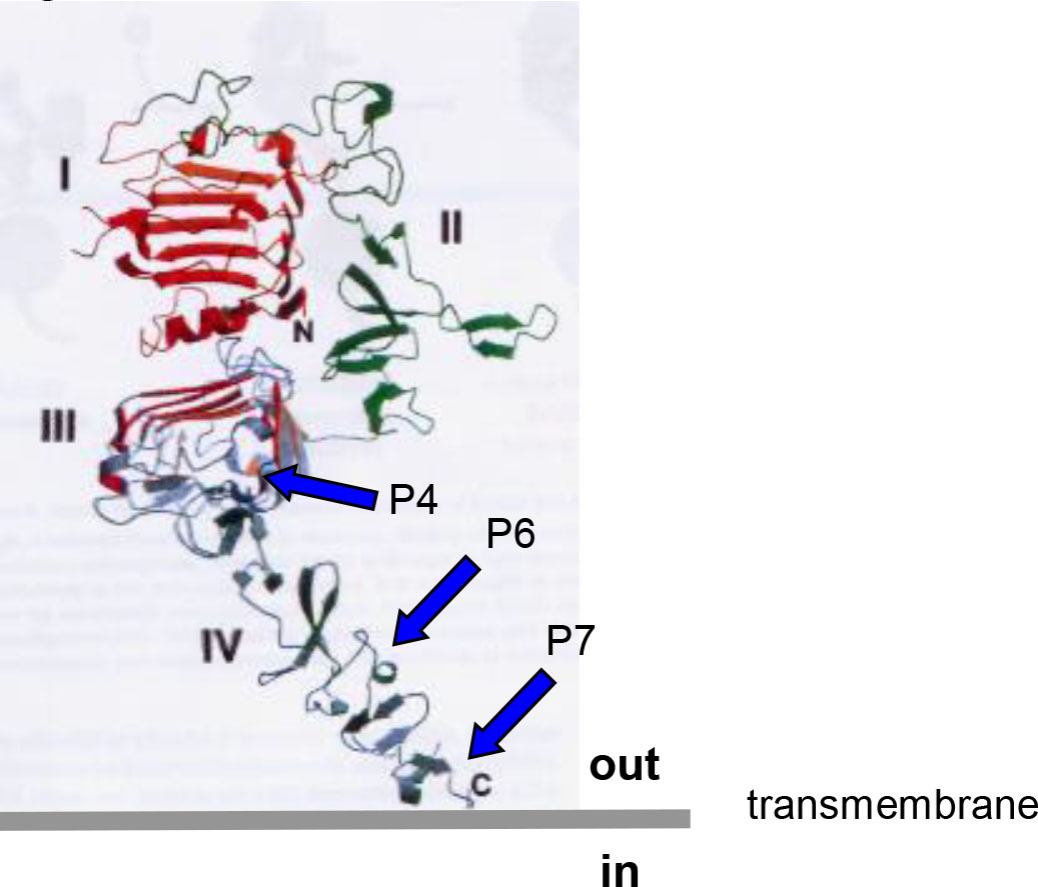
Structure and sequences of the extracellular domain of Her-2/neu (UniProt #P04626).

The Wiedermann Group in Vienna has been developing different and evolving formulations of Her-2/neu vaccines for over 20 years (15–17, 20–23). HER-Vaxx (IMU-131), the proposed drug product, and P467-CRM197, the drug substance, in 10 mM phosphate buffered saline pH 7.4 (PBS) is under development as a therapeutic vaccine for the treatment of gastric cancer in patients with human epidermal growth factor receptor 2/neu (HER-2/neu)-overexpressing metastatic or advanced adenocarcinoma of the stomach or gastroesophageal junction, referred to as advanced cancer of the stomach. P467-CRM197 contains a single collinear peptide antigen composed of 3 individual linear B-cell epitope peptide sequences originally selected from the extracellular domains III and IV of HER-2/neu that induce the patient’s own B cells to produce endogenous anti-HER-2/neu antibodies (20). See **Figure 1**.

The published results from a number of studies indicated that the optimal Her-2/neu epitopes for the proposed therapeutic vaccine were P4, P6, and P7 (i.e., P467), that CRM197 (a nontoxic diphtheria toxin variant) was the better of the conjugating carrier proteins evaluated and used to produce P467-CRM197 (the designated drug substance), and that Montanide ISA 51 VG Sterile was a good adjuvant for preparing the emulsion formulation (IMU-131 [the proposed drug product] emulsified to prepare the dosage form, HER-Vaxx) to be administered intramuscularly (IM) (16).

P467-CRM197 has been optimized to provide a vaccine that produces a stronger and more rapid polyclonal antibody response than previous constructs (16), i.e., a single peptide antigen (P467) based on P4, P6, and P7, allowing a simplified manufacturing process. First, only 1 clinical grade batch of peptide (P467) needs to be produced and qualified rather than 3 separate peptide batches (P4, P6, and P7). Second, compatibility problems in the formulation process caused by different physicochemical properties of the 3 peptides are circumvented. Third, equal dosing of the 3 epitopes in the P467 construct is guaranteed by stoichiometry within a single peptide.

Extensive immunological studies were conducted in mice (16) that included the following.

- Evaluation of immunogenicity:

◦ Humoral responses (IgG, IgG1, IgG2a)
◦ Cellular responses (interleukin [IL]-2, IL-5, IFN-γ)

- Comparison of different examined doses and adjuvants (Montanide vs alum)
- Characterization of splenocytes populations; IFN-γ production on single cell level

In summary, P467-CRM197 (administered IM and SC as HER-Vaxx) has been shown to elicit both humoral and cellular immune responses. The cellular response was relatively mild and was mainly attributed to the carrier protein CRM197. CRM197 is an established carrier used in authorized vaccine products distributed worldwide (e.g., Prevnar13, Pfizer and Vaxneuvance, MSD). Given these data, a strong cellular response (potentially including the induction of a cytokine storm) to the whole product would not be expected.

**Figure 2:**
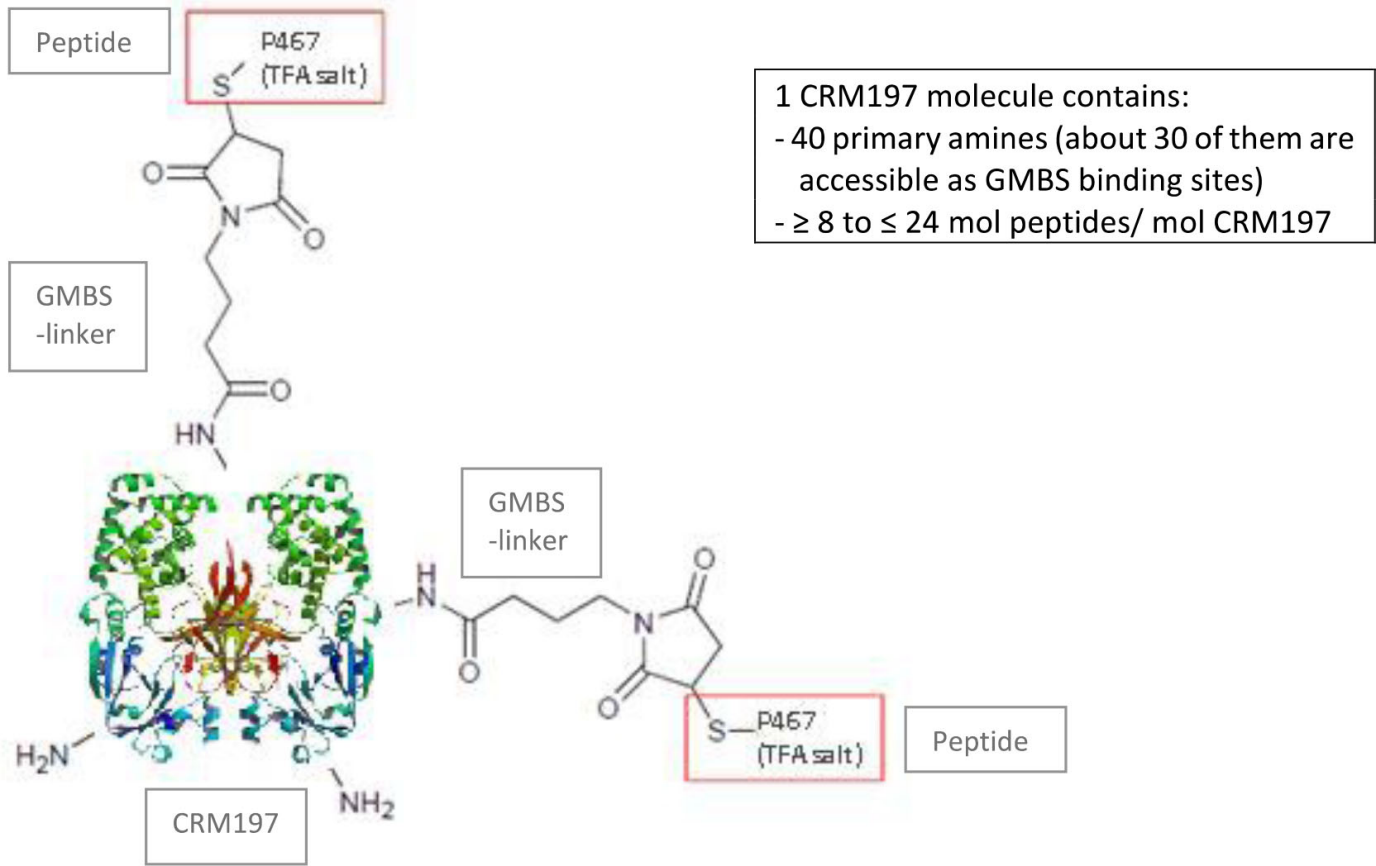
Schematic structure of P467-CRM197.

The conjugate P467-CRM197 (defined as active substance/drug substance) consists of the peptide P467 (see derivation of P467 from extracellular domain of Her-2 in **Figure 1**) which is covalently conjugated *via* GMBS to the high molecular carrier protein CRM197 (cross reacting material 197; non-toxic variant of diphteria toxin) as shown in **Figure 2**, manufactured under cGMP by piCHEM Forschungs- und Entwicklungs GmbH (Graz, Austria) and sterile filled and finished by Baccinex SA (Courroux, Switzerland). In detail, the peptide P467 is coupled through the sulfhydryl group of its N-terminal Cysteine to GMBS (4-Maleimidobutyric acid N-Hydoxysuccinimide ester), a heterobifunctional cross-linking reagent with amine and sulfhydryl reactivity. GMBS is coupled *via* its N-hydroxysuccinimide ester group to primary amines of carrier protein CRM197 to form stable amide bonds. Theoretically, there are 40 primary amines available per CRM197 molecule (39 x Lysine, 1 x N-terminus), but not all of them are accessible for the reaction with GMBS. Based on experience the ratio of CRM197 to GMBS is in the best case about 1:30. The peptide/CRM197 ratio (complies to the ratio peptide P467/conjugate P467-CRM197) is specified to be ≥ 8 mol/mol to ≤ 24 mol/mol. Stability data are available for P467-CRM197 for 48 months at both the normal storage temperature of -20°C and accelerated temperature of +5°C. All of the results met specifications.

The components of IMU-131 are supplied in 2 vials: an aqueous solution of the peptide antigen conjugated to the carrier protein CRM197 (200 µg per vial P467-CRM197 in saline (1mL)) and the adjuvant Montanide ISA 51 VG (SEPPIC, Paris France). The 2 components are mixed in a 1:1 ratio to form a water-in-oil emulsion of the final dosage form (HER-Vaxx) for administration by intramuscular (IM) injection. The concentration of the dose is constant (100 µg/mL) with the actual dose delivered based on volume injected.

### Preclinical studies including pharmacological and GLP toxicology and safety pharmacology

The GLP studies were conducted in compliance with the United States FDA GLP Regulations (21 CFR Part 58), 05-Oct-1987. Charles River SOPs (for activities conducted at Charles River).

### Safety pharmacology

#### (1) P467-CRM197: Cardiovascular and respiratory assessment following intramuscular administration to conscious, radiotelemetry-instrumented beagle dogs

The objective of this GLP-compliant study was to assess the potential acute effects of P467-CRM197 (IMU-131 administered IM as HER-Vaxx) on arterial blood pressure, heart rate, body temperature, lead II electrocardiogram (ECG), and respiratory parameters in conscious, radiotelemetry-instrumented male beagle dogs. This study also evaluated the potential effects of anti-P467-CRM197 antibodies on the evaluated cardiovascular and respiratory parameters in assessments made after a 2-week recovery period, when P467-CRM197 was expected to have been completely cleared from the body.

The test article, P467-CRM197, was a peptide vaccine comprising a peptide antigen that included 3 B-cell epitopes to Her-2/neu ECD that were covalently linked to a carrier protein (CRM197). The peptide vaccine (IMU-131) was formulated 1:1 (v/v) with a vaccine adjuvant, Montanide ISA 51 VG, to form HER-Vaxx. The vaccine adjuvant alone and IMU-131 at 0.26 mg/mL and emulsified with the vaccine adjuvant to produce the P467-CRM197 dose of 52.0 µg/dose were administered by IM injection (200 µL per injection) to 6 male beagle dogs (see **Table 1** for study group assignments). The same 6 dogs received the vaccine adjuvant dose (Group 1) during the pretest period followed by emulsified IMU-131 (Group 2) on Study Days 0, 13, and 27. Heart rate, arterial blood pressure (systolic, diastolic, and mean arterial pressure), pulse pressure, body temperature, ECG waveforms (from which the ECG intervals PR, QRS, QT, and heart rate-corrected QT [QTcV] were derived), and respiratory data (respiratory frequency, tidal volume, and minute volume) were collected continuously for approximately 24 hours following administration of vaccine adjuvant (pretest), the test article (Study Day 0), or on Study Day 41 (14 days following the last dose on Study Day 27). Clinical observations were performed at approximately 4 and 24 hours after dosing. Injection site observations were performed prior to dosing, approximately 1 and 24 hours after each day of dosing, and on Study Day 41. Blood samples were collected prior to dosing and on Study Days 27 and 41 for clinical pathology evaluations. In addition, blood samples were collected prior to dosing and on Study Days 27 and 41 for anti-P467-CRM197 antibody analyses.

**Table 1:** Study group assignments.

Group	Treatment	DoseLevel (µg/dose)	Dose Concentration(µg/µL)	Dose Volume ^a^ (µL)	Numberof Males ^b^
1	Adjuvant ^c^	0	0	200	6
2	P467-CRM197 ^d^	52.0	0.26	200	6

a The total dose volume administered per dose (200 µL) was split approximately equally across 2 different injection sites, such that approximately half of the total dose volume (100 µL) was administered at one site and the remaining volume was administered at the other site. On dosing days, treatment administration across each dose site for an individual animal occurred at approximately the same time.

b The same 6 animals received each dose with the adjuvant dose (Group 1) being administered during the pretest period followed by the test article (Group 2) on study days 0, 13, and 27.

c The adjuvant was Montanide ISA 51 VG Sterile; the adjuvant control treatment administered to Group 1 animals included 100 µL of Montanide ISA 51 VG Sterile and 100 µL of 10 mM phosphate buffered saline, pH 7.4, in a given administered dose.

d P467-CRM197 treatment included equal parts (1:1) of P467-CRM197 (in 10 mM phosphate buffered saline, pH 7.4) and the adjuvant, Montanide ISA 51 VG Sterile.

Animals were dosed according to the following table:

IM administration of P467-CRM197 at 52.0 µg/dose did not cause any apparent adverse treatment effects on any of the cardiovascular or respiratory parameters evaluated or on any of the clinical pathology parameters. IM injection site reactions, which persisted throughout the study, were noted; however, these local tolerance findings either greatly lessened or resolved by Study Day 41. All predose serum samples were negative for anti-P467-CRM197 antibodies, and all collected post-dose serum samples were positive. Titer results for positive samples on Study Day 27 ranged from 604 to 2,508 and on Study Day 41 ranged from 490 to 2,274. These data for Study Day 41 suggest that in the presence of anti-P467-CRM197 antibodies, no apparent adverse effects were observed when compared to pretest results. Thus, anti-P467-CRM197 antibodies, like P467-CRM197, had no apparent adverse effects on any of the cardiovascular and respiratory parameters evaluated in this study in male beagle dogs.

Based on these results, the no-observed-effect level (NOEL) for cardiovascular and respiratory function in the beagle dog was considered to be 52.0 µg/dose of P467-CRM197 (IMU-131 administered IM as HER-Vaxx).

Serum samples collected from 6 beagle dogs administered IMU-131 (IM as HER-Vaxx) at 52.0 µg/dose on Study Days 0, 13, and 27 during a cardiovascular and respiratory safety pharmacology study (Study WIL-213502, CRL Ashland OH) were also tested in a HER-2+ gastric cell line proliferation inhibition study. The dog serum contained high levels of anti-P467-CRM197 antibodies and anti-Her-2 antibodies and was separately shown to cross react with both human and canine Her-2 (unpublished results, Mimotopes Pty Ltd, Australia). Pooled beagle sera reacted with both human and canine Her-2/ErbB2 HIS tagged proteins captured onto a nickel coated ELISA plate (see **Table 2**).

**Table 2:** Plate ELISA OD’s for each of the 3 Beagle Pools tested on each of the two Her2/ErbB2 proteins.

Beagle SERA	Protein 2 Human Her-2/ErbB2					Protein 2 Canine Her-2/ErbB2				
Dilution	Day-8	Day 27		Day 41		Day-8	Day 27		Day 41	
1/100	326	1584	1447	1673	1964	429	1282	1298	1665	1758
1/200	271	960	862	1134	1010	216	814	806	998	976
1/400	239	824	829	939	855	213	610	624	725	743
1/800	186	612	626	672	657	178	437	435	495	530
1/1600	155	427	425	436	411	154	295	290	330	349
1/3200	124	284	273	291	264	124	213	211	229	239
1/6400	102	179	182	206	165	97	150	149	163	165
1/12800	89	120	128	120	118	75	103	106	103	111

**Figure 3:**
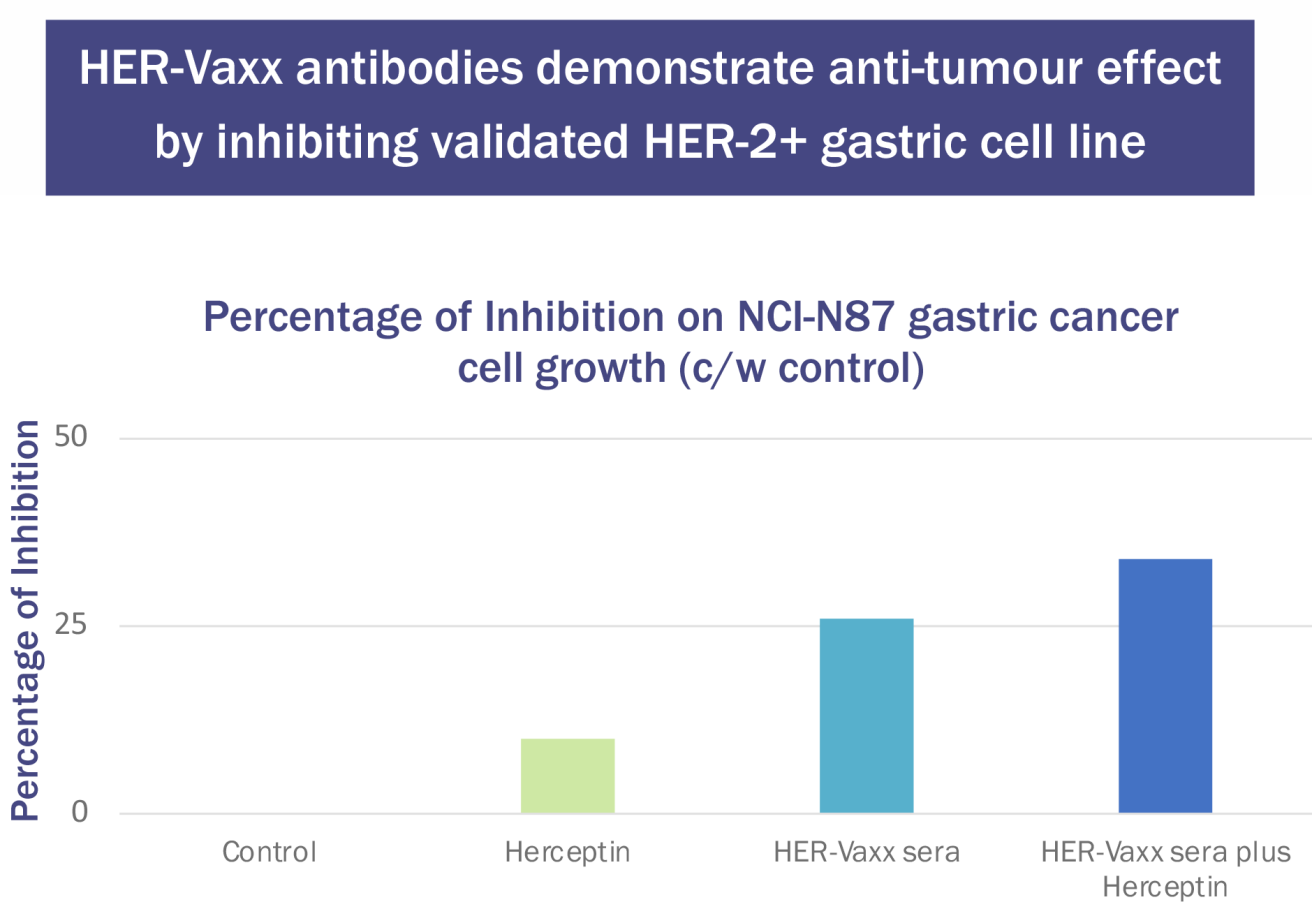
Effect of Herceptin (37.5μg/mL) and P467-CRM197 anti-Her-2 Antibody-Containing Sera (10μL/well) on NCI-N87 Cell Number.

These generated antibodies also inhibited the growth of the validated human Her-2+ gastric tumor cell line NCI-N87. The results were compared with those from Herceptin (trastuzumab). Tumour cell inhibition was higher after the second immunization of P467-CRM197 than after trastuzumab. When the serum was combined with trastuzumab, the inhibition was additive (**Figure 3**) (unpublished results, Charles River Laboratories, Morrisville NC).

#### (2) CNS safety pharmacology assessments made during a 28-day (once weekly) intramuscular injection toxicity study of p467-crm197 in Sprague Dawley rats with a 14-day recovery period

The objectives of this GLP-compliant, repeat-dose toxicology and immunogenicity study (unpublished results, Charles River Laboratories, Ashland OH) were to evaluate the potential toxicity of P467-CRM197 (IMU-131 emulsified with Montanide ISA 51 VG to form HER-Vaxx) when administered once weekly *via* IM injection to male and female Sprague-Dawley rats for 28 days (5 total doses; Study Days 0, 7, 14, 21, and 28), as well as to evaluate the recovery, persistence, or progression of any effects following a minimum of a 14-day recovery period (see **Table 1**). P467-CRM197 dose levels were 10.4, 31.2, and 52.0 µg/dose for Groups 3, 4, and 5, respectively. Group 1 rats received the vehicle (10 mM PBS) and Group 2 rats were administered vaccine adjuvant. IM dose volumes were 200 µL for rats in Groups 1, 2, and 5; 40 µL for rats in Group 3; and 120 µL for rats in Group 4. Each dose group consisted of 15 rats/sex. A minimum of 2 days following the last dose administration, 10 rats/sex/group were euthanized, and the remaining 5 rats/sex/group were euthanized following a minimum 14-day recovery period. Modified Irwin assessments and body temperature data (CNS safety pharmacology assessments) were recorded from 5 rats/sex/group at approximately 3 hours after dose administration on Study Days 0 and 21 and near the end of the recovery period (Study Day 37, when P467-CRM197 had been cleared from the body). The same 5 rats/sex/group were evaluated at each interval.

Passivity was noted for 2 Group 4 (31.2 µg/dose) male rats on Study Day 0. This finding, observed at a low frequency in male rats only at a single interval, was not considered related to P467-CRM197 administration because the effect was not observed in a dose-related manner. Decreased pinna reflex was noted in 1 Group 5 (52.0 µg/dose) female rat on Study Day 21. This finding was not considered related to P467-CRM197 administration because the effect was observed for only a single female rat at a single interval. No significant changes in body temperature in male rats were noted for any P467-CRM197 dose evaluated when compared to male rats in the vehicle or adjuvant control groups. A statistically significant, but minimal in magnitude (≤ 1.5°C), higher average body temperature was noted in the Group 3 (10.4 µg/dose) and Group 5 (52.0 µg/dose) female rats on Study Day 0 when compared to female rats in the vehicle control group and on Study Days 0, 21 (Group 5 only), and 37 when compared to the rats in the adjuvant control group. No statistically significant changes in average body temperature were noted in the Group 4 (31.2 µg/dose) female rats. Therefore, the modest changes in average body temperature in female rats in Groups 3 and 5 were not considered to be related to P467-CRM197.

The conclusion for these CNS safety pharmacology assessments on P467-CRM197 in male and female rats was that once weekly administration of P467-CRM197 (IMU-131 administered IM as HER-Vaxx) at 10.4, 31.2, or 52.0 µg/dose did not adversely affect the gross behavioral, physiological, or neurological state of the rat, or the body temperature, when evaluated on Study Days 0, 21, and 37. The Study Day 37 results, when P467-CRM197 had been cleared from the body, also indicated that the formed anti-P467-CRM197 antibodies, which were present in serum samples collected on Study Day 41, did not cause CNS adverse effects in any of the parameters evaluated.

### Repeat dose toxicology

#### (1) A 28-day (once weekly) intramuscular injection toxicity study of P467-CRM197 in Sprague Dawley Rats with a 14-day recovery period

The objectives of this GLP-compliant study (WIL-213502 unpublished results, Charles River Laboratories, Ashland OH) were to evaluate the potential toxicity of P467-CRM197 when administered IM as HER-Vaxx (a 1 to 1 emulsion formulation of IMU-131 and Montanide ISA 51 VG Sterile) to male and female Sprague Dawley rats for 28 days (5 total doses; Study Days 0, 7, 14, 21, and 28), as well as to evaluate the recovery, persistence, or progression of any effects following a minimum of a 14-day recovery period. **Table 3** summarizes the rat group assignment and P467-CRM197 dose levels for this study. A minimum of 2 days following the Study Day 28 dose administration, 10 rats/sex/group were euthanized; the remaining 5 rats/sex/group were euthanized following a minimum 14-day recovery period.

**Table 3:** Group Assignments for WIL-213502.

Group Number	Treatment	P467-CRM197 Dose Level (µg/dose)	Dose Volume (µL)	Number of Rats^a^	
				Males	Females
1	Vehicle ^b^	0	200	15	15
2	Adjuvant ^c^	0	200	15	15
3	P467-CRM197 ^d^	10.4	40	15	15
4	P467-CRM197 ^d^	31.2	120	15	15
5	P467-CRM197 ^d^	52.0	200	15	15

a 10 rats/sex/group were assigned to the primary necropsy; the remaining 5 rats/sex/group were assigned to a minimum 14-day recovery period.

b 10 mM phosphate buffered saline, pH 7.4 (PBS).

c The vaccine adjuvant was Montanide ISA 51 VG which was administered to Group 2 rats after emulsification of 100 µL of Montanide ISA 51 VG and 100 µL of PBS.

d P467-CRM197 treatment included equal parts (1:1 v/v) of P467-CRM197 in PBS (IMU-131) and the vaccine adjuvant.

In addition, prior to dosing on Study Day 28, and on the day of recovery necropsy (Study Day 43 or 44), serum samples were collected during acclimation and analyzed for the formation of anti-P467-CRM197 antibodies (using the validated enzyme-linked immunosorbent assay [ELISA] method, summarized below) and the positive samples were titrated.

For toxicology assessments, all rats were observed twice daily for mortality and moribundity. Clinical examinations were performed twice daily on the days of dose administration, at the time of dose administration, and at 1 to 2 hours after dose administration. IM injection site observations were performed prior to dose administration, at approximately 1 to 2 hours after dose administration, on the day of the primary necropsy (only rats scheduled for necropsy), weekly (± 2 days) during the recovery period, and on the day of the recovery necropsy. Detailed physical examinations and individual body weights were performed prior to and on the day of randomization, weekly prior to the initiation of dose administration, weekly during the study period, and on the days of the scheduled necropsies. Modified Irwin test assessment (central nervous system safety pharmacology evaluations) and body temperature data were recorded from 5 rats/sex/group at approximately 3 hours after dose administration on Study Days 0 and 21 and near the end of the recovery period (Study Day 37). Ophthalmic examinations were performed during the acclimation period and on Study Day 25. Clinical pathology parameters (hematology, coagulation, serum chemistry, and urinalysis) were evaluated for all rats assigned to the primary and recovery necropsies. Complete necropsies were conducted on all rats, and selected organs were weighed at the scheduled necropsies. Selected tissues were examined microscopically from all rats in Groups 1, 2, and 5. In addition, gross lesions and IM injection sites were examined from all rats in Groups 3 and 4 at the primary necropsy and from all rats at the recovery necropsy.

Using the validated analytical chemistry method, the dosing formulations were analyzed for P467-CRM197 content and each of the 3 dosing formulations were found to contain 94.0% to 100% of the P467-CRM197 formulated levels, which was within the acceptance range of target concentrations for emulsions (85% to 115%) and were uniform. No P467-CRM197 was detected in the analyzed phosphate buffered vehicle (Group 1) or vaccine adjuvant (Group 2) formulations.

All rats survived to the scheduled necropsies. No adverse test article-related clinical or IM injection site observations were noted. No test article-related adverse effects on body weights, food consumption, and Irwin assessments or on clinical pathology (hematology and coagulation, serum chemistry, and urinalysis) parameters were noted. No apparent test article-related ophthalmic, organ weights, or macroscopic findings were observed.

At necropsy, the IM injection sites or adjacent areas of tissue of a minimal number of female rats in the vaccine adjuvant control and test article-treated (31.2 µg/dose and 52.0 µg/dose) groups had grossly visible deposits of a white precipitate. Histologic examination of the injection sites from these rats commonly revealed clear vacuoles of various sizes that were consistent with the adjuvant material. An inflammatory cell infiltration at the IM injection sites consisted primarily of mononuclear cells and histiocytes, with minor populations of neutrophils, eosinophils, and plasma cells. The incidence pattern of the inflammatory cell infiltration suggested this finding was associated with the adjuvant, with no apparent increase in the incidence or severity of the inflammation being attributable to the presence of P467-CRM197. Plasma cells were more commonly noted in the IM injection sites of rats that received P467-CRM197 when compared to injection sites of rats that received the vehicle or adjuvant alone. No evidence of systemic toxicity associated with IM administration of P467-CRM197 was noted. None of the observed histological changes were considered to be adverse or related to P467-CRM197 administration. The IM injection sites of recovery group rats commonly had deposits of vacuolar material with associated inflammatory cell infiltrations, but the inflammatory cell infiltrates were less pronounced than those seen in the primary necropsy rats. This observation suggested that resolution of the IM injection site lesions was underway but incomplete at the time of the recovery necropsy.

#### (2) Detection of anti-P467-CRM197 antibodies in Sprague Dawley Rats serum

Anti-P467-CRM197 antibodies were detected in rat serum collected prior to dose administration on Study Day 28 (last administered dose) and on the day of the recovery necropsy from rats in the P467-CRM197 10.4, 31.2, and 52 µg/dose groups. For male rats, the average anti-P467-CRM197 antibody titers in the 10.4, 31.2, and 52 µg/dose groups were 2148, 3314, and 3017, respectively, on Study Day 28, and 1893, 3306, and 2673, respectively, at the end of the recovery period. For female rats, the average anti-P467-CRM197 antibody titers in the 10.4, 31.2, and 52 µg/dose groups were 1744, 3724, and 4140, respectively, on Study Day 28, and 1229, 7896, and 10,395, respectively, at the end of the recovery period. On Study Day 28, average antibody titers for male and female rats in the P467-CRM197 10.4, 31.2, and 52 µg/dose groups were generally similar at corresponding dose levels, while on Study Day 43/44, average antibody titers for female rats in the P467-CRM197 31.2 and 52 µg/dose groups were higher than those noted for the male rats at the same dose levels at the same evaluation intervals. Generally, antibody titers noted for male and female rats in the P467-CRM197 31.2 and 52 µg/dose groups at both evaluation intervals were higher than those noted for male and female rats in the 10.4 µg/dose groups. When values for male and female rats were combined, the average antibody titers on Study Day 43/44 increased in relationship to the P467-CRM197 dose. Anti-P467-CRM197 antibodies were not detected in serum from any rat in the vehicle control or vaccine adjuvant control groups at any evaluation interval.

Based on the results and findings of this study, IM injections of HER-Vaxx (1:1 [v/v] IMU-131and Montanide emulsion formulations) at P467-CRM197 dose levels of 10.4, 31.2, and 52.0 µg/dose once weekly for 28 days (5 total doses; Study Days 0, 7, 14, 21, and 28) to male and female Crl : CD(SD) rats were well tolerated and no apparent adverse test article-related findings were noted at any dose level tested. Anti-P467-CRM197 antibodies were detected (and titered) in serum samples collected on Study Days 28 and 43/44 from male and female rats at each P467-CRM197 dose level. Therefore, the no-observed-adverse-effect level (NOAEL) for this study in male and female rats was considered to be 52.0 µg/dose for P467-CRM197 administered IM as HER-Vaxx.

These findings suggest that P467-CRM197 has an acceptable safety profile in male and female rats. This rat safety profile is considered sufficient to support the clinical development of HER-Vaxx (the dosage form) as a therapeutic cancer vaccine for treatment of patients with advanced stomach cancer.

### Preclinical support for combination therapy targeting Her-2 and PD1

The investigation of HER-Vaxx plus pembrolizumab is planned in Arm 2 of the nextHERIZON study. Nonclinical data has demonstrated a combination of Her-2 and PD1 vaccines resulted in synergistic tumor growth inhibition in an Her-2+ syngeneic mouse model (24). It has also been observed that the immune system substantially contributes to the therapeutic effects of Her-2-targeted antibodies and the treatment with trastuzumab may increase tumor expression of PD-L1, which is potentially driving resistance to trastuzumab (14). These observations support the treatment combination with an immune checkpoint inhibitor.

#### (1) Active immunization combining mouse PD1-derived vaccine together with HER-Vaxx potentiates the anti-tumor effect *in vivo*

**Figure 4:**
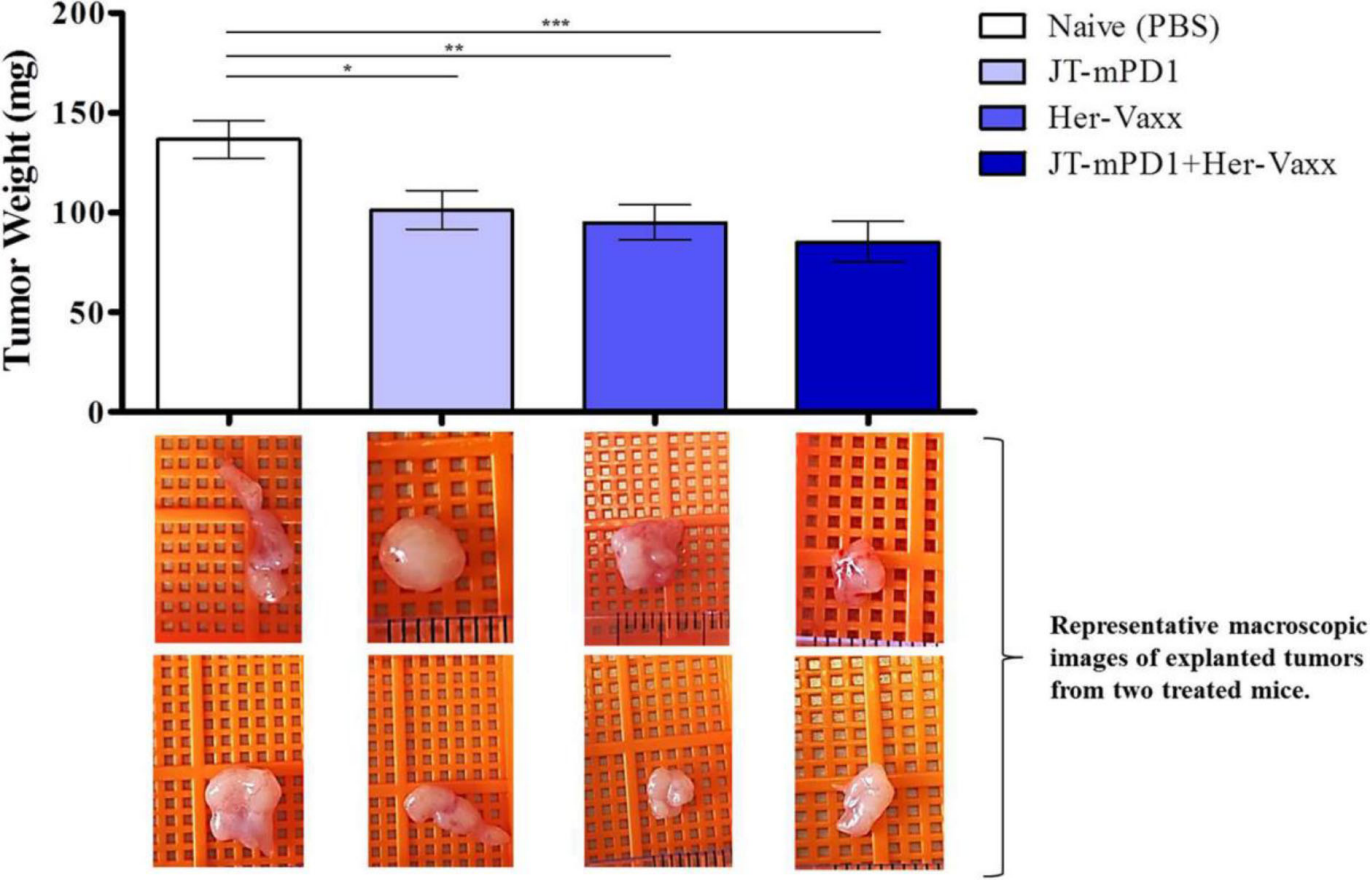
Reproduced with permission from (25) Results of tumor growth inhibition resulting from immunization with PD1-derived vaccine (JT-mPD1), HER-Vaxx or a combination of both in a syngeneic Her-2/neu mammary tumor mouse model. The results were representative of repeated experiments. Significant differences are indicated by asterisks (*p < 0.05, **p < 0.01, ***p < 0.001). All analyses were done by Stata 13.1 (StataCorp, TX, USA), and graphs were prepared by GraphPad Prism 7 (GraphPad Software, CA, USA). For all statistical tests, a value of *p* < 0.05 was considered significant. Figure presented with permission from Frontiers in Immunology.

In this pharmacology effectiveness study supporting the combination of HER-Vaxx with a PD1 checkpoint, the mouse PD1- derived vaccine (JT-mPD1, 50 μg/dose) in combination with HER-Vaxx vaccine (25 μg/dose), enhanced the anti-tumor effect *in vivo* in the mouse syngeneic tumor model (D2F2/E2 cells (BALB/c mouse mammary carcinoma cells expressing HER-2/neu) as previously reported (25). As shown in **Figure 4** (reproduced from (25), when mice were dual immunized with a combination of mouse PD1-derived vaccine combined with HER-Vaxx vaccine, tumor growth inhibition measured by tumor weight was significantly potentiated compared to the effect seen in the mice immunized with each vaccine alone.

### HER-Vaxx clinical trial data: what’s “next”

#### (1) Phase 1 and 2 trials

Clinical support for HER-Vaxx is based on 50 patients (14 in Phase 1b and 36 in Phase 2) diagnosed with metastatic or advanced Her-2/neu-overexpressing, gastric or GEJ adenocarcinoma enrolled in the HER-Vaxx (IMU-131) clinical development program (IMU.ACS.001), of which 33 patients (14 in Phase 1b and 19 in Phase 2) were dosed with HER-Vaxx (IMU-131). The preliminary immunology and clinical response data from this program are promising. Safety data to date indicate that HER-Vaxx (IMU-131) is well tolerated with no significant local or systemic reactions, and no need for pretreatment or for modification to the dose or treatment schedule due to safety. The safety and immunological data from Phase 1b supported a dose of 50 µg peptide P467 antigen equivalent conjugated to CRM197 (IMU-131) in a Montanide emulsion (HER-Vaxx) as a 0.5 mL injection on Days 0, 14, and 35 followed by a booster 42 days later and subsequent boosters every 63 days (15). The 50 µg dose induced consistently high P467-specific antibodies and HER2/neu-specific antibodies with an observed correlation of titer levels with clinical response (15). Higher doses of HER-Vaxx (IMU-131) are being investigated as part of a Phase 2 extension to explore whether an increased dose will produce earlier and stronger development of HER2/neu antibodies.

The Phase 2 part of the ongoing randomized controlled HERIZON study (IMU.ACS.001) is designed to assess safety and efficacy of HER-Vaxx (IMU-131) plus chemotherapy (cisplatin and 5-FU or capecitabine, or oxaliplatin and capecitabine) in patients with advanced or metastatic GC/GEJ cancer. At the time of interim analysis, this study showed an overall survival (OS) benefit with a hazard ratio (HR) of 0.418 (2-sided 80% CI: 0.186, 0.942). Safety, in terms of hematological and non-hematological adverse events, was shown to be similar between the treatment arms with no significant vaccination-related toxicity (26). Final analysis in the randomized Phase 2 trial showed statistically significant overall survival Hazard Ratio (HR) of 0.585 (80% 2-sided CI: 0.368, 0.930). HER-Vaxx showed a reduced risk of death of 41.5% in the HER-Vaxx plus chemotherapy group compared to chemotherapy alone. The median overall survival (OS) for patients receiving HER-Vaxx plus chemotherapy was 13.9 months, compared to 8.3 months in patients treated with chemotherapy alone. The Phase 2 trial confirms a favourable survival outcome with no added toxicity for HER-Vaxx combined with standard-of-care (SOC) chemotherapy over chemotherapy alone. Final analysis is pending on completion of the study.

#### (2) Combination of HER-Vaxx plus pembrolizumab – nextHERIZON

The proposed new clinical study, nextHERIZON (IMU.131.203), will investigate two different treatment regimens in patients who are confirmed Her-2/neu-overexpressing after progression on a previous trastuzumab-containing treatment regimen. The objective for combining HER-Vaxx (IMU-131) with chemotherapy (ramucirumab plus paclitaxel) is to provide an alternative treatment to Enhertu^®^. In contrast to trastuzumab-deruxtecan (Enhertu^®^), HER-Vaxx (IMU-131) can be added to standard-of-care chemotherapy and continue as a maintenance after chemotherapy is terminated. Data from the ongoing Phase 2 study indicate treatment with HER-Vaxx (IMU-131) is well tolerated with no added toxicity, dose-limiting toxicity, or immune-related adverse events.

The title of the clinical study, IMU.131.203, is called “nextHERIZON: Phase 2 study, exploring treatment with HER-Vaxx in combination with chemotherapy or HER-Vaxx with pembrolizumab in previously trastuzumab treated metastatic Gastric or GastrO-esophageal junction cancer Patients who have progressed under this treatment.” ClinicalTrials.gov Identifier: NCT05311176.

This is a Phase 2, signal-generating, open-label, 2-arm, non-randomized study in adult patients with metastatic HER2/neu-overexpressing gastric cancer (GC) or GEJ adenocarcinomas are planned to be conducted at sites in Australia and the US. Study objectives are to assess safety and efficacy, and to evaluate HER2/neu antibodies in patients treated with HER-Vaxx (IMU-131) who have progressed following trastuzumab treatment.

Support for the combination of HER-Vaxx with pembrolizumab is found in preclinical data that recently documented active immunization with HER-Vaxx prevents lung metastases formation with concomitant increased PD-L1 expression in a Her-2/neu positive lung metastases mouse model (27). A combination therapy targeting both Her-2/neu and the PD1/PD-L1 axis may be used clinically and synergistically to treat metastatic Her-2+ cancers.

The elucidation of various molecular pathways involved in the development of GC has led to a multitude of studies of targeted therapies. Following the success of the ToGA trial, agents such as those targeting the vascular endothelial growth factor (VEGF) angiogenesis pathway have been investigated. Ramucirumab is a human IgG1 monoclonal antibody receptor antagonist designed to bind to the extracellular domain of VEGFR-2. The phase III RAINBOW trial evaluated the efficacy and safety of ramucirumab plus paclitaxel vs placebo plus paclitaxel in patients with locally advanced or metastatic gastric or GE junction adenocarcinoma who have progressed after first-line chemotherapy (28). OS was significantly longer in the ramucirumab plus paclitaxel group than in the placebo plus paclitaxel group (median 9·6 months [95% CI: 8·5,10·8] vs 7·4 months [95% CI: 6·3,8·4], hazard ratio 0·807 [95% CI: 0·678, 0·962]; p=0·017). PFS was significantly increased in the treatment group compared to the control group (4.4 months [95% CI: 4.2, 5.3] vs 2.9 months [95% CI: 2.8,3.0], hazard ratio 0·64 [95% CI: 0·54, 0·75]; p<0·001). Confirmed ORR was 28% (95% CI: 23, 33) in the ramucirumab plus paclitaxel arm compared with 16% (95% CI: 13, 20) for those receiving placebo plus paclitaxel. Ramucirumab plus paclitaxel is now approved as a second-line treatment option for patients with metastatic gastric cancer, who failed first-line treatment with platinum- and fluoropyrimidine-based combinations or trastuzumab in combination with cisplatin and 5-fluorouracil/cisplatin and capecitabine.

**Figure 5:**
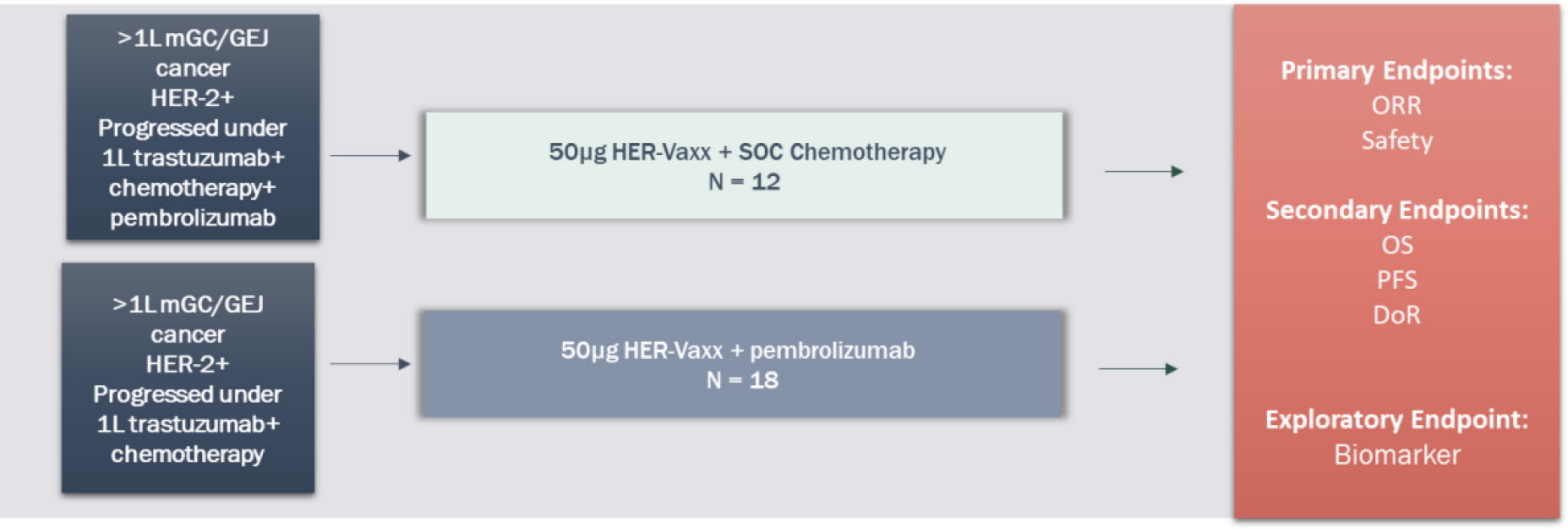
nextHERIZON Synopses.

The study includes 2 treatment arms that will be analyzed independently using a 2-stage design. Arm 1 is 50 µg HER-Vaxx (IMU-131) + chemotherapy (ramucirumab plus paclitaxel) and Arm 2 is 50 µg HER-Vaxx (IMU-131) + 200 mg pembrolizumab Q3W. See **Figure 5**.

The intended population is approximately 30 adult GC/GEJ cancer patients who have confirmed HER2/neu overexpression since progression on trastuzumab by tumor biopsy (post-progression fresh or archival tissue, or post-progression pathology report). Patients who have received an immune checkpoint inhibitor (ICI) previously will preferentially be enrolled in Arm 1 and patients who are naïve to ICI treatment will preferentially be enrolled into Arm 2. Patients who have had chemotherapy only treatment after progression on trastuzumab (± ICI) are eligible for this study whereas patients who have received trastuzumab-deruxtecan (Enhertu^®^) or any anti-Her-2 targeted therapy other than trastuzumab are ineligible.

Patients may continue treatment with HER-Vaxx (IMU-131) until progression after termination of chemotherapy (expected a minimum of 3 cycles) or pembrolizumab (expected until progression).

## Conclusion

These preclinical and early Phase 1 and 2 clinical findings suggested that HER-Vaxx has an acceptable safety and efficacy profile. The profile is considered sufficient to support the clinical development of HER-Vaxx as a therapeutic cancer vaccine for treatment of patients with advanced stomach cancer.

Combining HER-Vaxx and pembrolizumab will investigate this treatment in patients who have previously progressed under trastuzumab and chemotherapy but have not been treated with a checkpoint inhibitor in 1L. Patients whose tumor is still Her-2/neu-overexpressing will likely be sensitive to continued targeted treatment against Her-2/neu-overexpressing GC/GEJ cancer and may benefit from combination with HER-Vaxx and pembrolizumab due to the earlier described upregulation of PD-L1 and potential synergy between the these agents. Subsequently the treatment may lead to an overall survival benefit.

An issue with immunotherapy treatments combining targeted monoclonal antibodies such as those targeting tumor-associated antigens such as trastuzumab (Her-2) and immune checkpoints such as pembrolizumab (PD1), nivolumab (PD1) and ipilimumab (CTLA4) is that their combination can add toxicity and cost of treatment. B cell vaccines such as HER-Vaxx have demonstrated excellent safety profiles and the cost of manufacture of peptide vaccines are less expensive. Combined with less frequent and patient friendly dosing regimens, active immunizations with B cell vaccines are a potentially superior alternative to standard of care passive infusion of monoclonal antibodies.

In summary, immuno-oncology combinations are what is driving value presently in oncology drug development. What is needed for improved response rates and outcomes for patients are combinations that (a) combine without increasing toxicity, (b) combine with minimal cost increase, and (c) combine for better response rate and efficacy. The B cell active immunization strategy positions B cell vaccines to provide solutions for these three criteria and our preclinical and clinical data supports future combinations.

## Author contributions

NE, AG, and UW: conception and design. NE, AG, JT, EG-S, and UW: development of methodology. NE, AG, JT, EG-S, and UW: acquisition of data (provided animals, acquired and managed patients, provided facilities, etc). NE, AG, JT, EG-S, and UW: analysis and interpretation of data (e.g., statistical analysis, biostatistics, computational analysis). NE and JT: writing, review, and/or revision of the manuscript, writing and finalizing. NE, AG, JT, EG-S, CZ and UW: reviewing and commenting. All authors contributed to the article and approved the submitted version.
